# Respiratory Viral Infection Patterns in Hospitalised Children Before and After COVID-19 in Hong Kong

**DOI:** 10.3390/v16111786

**Published:** 2024-11-17

**Authors:** Jason Chun Sang Pun, Kin Pong Tao, Stacy Lok Sze Yam, Kam Lun Hon, Paul Kay Sheung Chan, Albert Martin Li, Renee Wan Yi Chan

**Affiliations:** 1 Department of Paediatrics, Faculty of Medicine, The Chinese University of Hong Kong (CUHK), 6/F Lui Che Woo Clinical Sciences Building, Prince of Wales Hospital, Shatin, New Territories, Hong Kong, China; jason@link.cuhk.edu.hk (J.C.S.P.); marstao@cuhk.edu.hk (K.P.T.); ehon@cuhk.edu.hk (K.L.H.); albertmli@cuhk.edu.hk (A.M.L.); 2 Hong Kong Hub of Paediatric Excellence, The Chinese University of Hong Kong, 8/F Research Office, Tower A, Hong Kong Children’s Hospital, 1 Shing Cheong Road, Kowloon Bay, Kowloon, Hong Kong, China; 3 Laboratory for Paediatric Respiratory Research, Li Ka Shing Institute of Health Sciences, Rm 201, Li Ka Shing Medical Sciences Building, The Chinese University of Hong Kong, Prince of Wales Hospital, Shatin, New Territories, Hong Kong, China; 4 Department of Microbiology, Faculty of Medicine, The Chinese University of Hong Kong, Shatin, New Territories, Hong Kong, China; paulkschan@cuhk.edu.hk; 5 SH Ho Research Centre for Infectious Diseases Faculty of Medicine, Rm 207, 2/F, S.H. Ho Research Centre for Infectious Diseases, JC School of Public Health Building, Prince of Wales Hospital, Shatin, New Territories, Hong Kong, China

**Keywords:** co-infection of respiratory viruses, paediatric respiratory virus epidemiology, age-specific responses, post-COVID-19

## Abstract

The study highlights the significant changes in respiratory virus epidemiology following the lifting of COVID-19 restrictions. Method: In this single-centre retrospective study, the virological readouts of adenovirus (AdV), influenza virus A (IAV), influenza virus B (IBV), parainfluenza viruses (PIV) 1, 2, 3, 4, respiratory syncytial virus (RSV), and coupled enterovirus and rhinovirus (EV/RV) were extracted from the respiratory specimens of paediatric patients in Hong Kong from January 2015 to February 2024. The subjects were stratified into five age groups. Results: The study included 18,737 and 6001 respiratory specimens in the pre-COVID-19 and post-COVID-19 mask mandate period, respectively. The mean age of hospitalised patients increased from 3.49 y ± 0.03 y to 4.37 y ± 0.05 y after the COVID-19 lockdown. The rates of single-virus infection and co-infection were significantly higher in the post-COVID-19 mask mandate period. The odds ratio for AdV for all age groups (OR: 4.53, 4.03, 2.32, 2.46, 1.31) and RSV in older children from 3 years old and above (OR: 1.95, 3.38, *p* < 0.01) were significantly elevated after the COVID-19 outbreak. Conclusions: Our findings suggest that public health measures to contain COVID-19 may have unintended consequences on children’s natural exposure and immunity to other respiratory viruses, potentially increasing their morbidity in the post-pandemic era.

## 1. Introduction

Hong Kong, a city that stands out for its implementation of a zero-COVID policy [1], has provided a unique setting for our study. This policy, which included strict border control, mandatory quarantine, widespread testing, contact tracing, and universal masking, led to a distinct period in Hong Kong with minimal respiratory virus circulation, except for SARS-CoV-2. This unique environment served as a natural experiment via which to assess whether the disappearance of respiratory viruses would be associated with a change in respiratory virus epidemiology when the population resumed their normal routine after the removal of the mask mandate on 1 March 2023.

Our focus on children is crucial due to their ongoing lung and immunity development. Exposure to viruses at different age windows can influence the development of immune memory and, consequently, their responses to the same virus compared to those regularly exposed during childhood. The COVID-19 pandemic can potentially alter the intensity of respiratory virus exposure in certain age groups. Therefore, an age-stratified analysis is vital to determine if a specific virus is more closely linked to the vulnerability of a particular age group. This underscores the potential adverse effects on children’s health, the increased burden on the healthcare system, and the necessity for adjustments in hospitalisation and vaccination strategies due to the alternations in virus prevalence.

## 2. Materials and Methods

To investigate this, our study analysed the prevalence patterns of respiratory viruses in hospitalised paediatric patients in Hong Kong, leveraging the well-defined periods of stringent ‘zero-COVID’ policies. Our study extracted the paediatric hospitalisation records of those below 18 years old at the Prince of Wales Hospital from January 2015 to February 2024. We included subjects presented with respiratory symptoms and collected nasopharyngeal aspirate (NPA) or swab (NPS) specimens from their upper airways that were sent for the multiplex PCR detection of respiratory viruses during admission. The array of viruses assessed in this study included adenovirus (AdV), influenza virus A (IAV), influenza virus B (IBV), parainfluenza viruses (PIV) 1, 2, 3, 4, respiratory syncytial virus (RSV), and coupled enterovirus and rhinovirus (EV/RV). We classified the era from January 2015 to December 2019 as the ‘pre-COVID-19 era’, that from January 2020 to February 2023 as ‘COVID-19 lockdown’, and that from March 2023 to February 2024 as the ‘post-COVID-19 mask mandate period’. The prevalence of individual viruses was defined by the number of positive tests among all the virology tests performed, with all samples retained as the denominator to address the fluctuations in sample size over the study period. Samples with uncertain results were excluded from the analysis. There was a change from testing NPA (2015–2019) to NPS (2020 onwards) due to the need for infection control, as NPA collection is considered an aerosol-generating procedure compared to NPS. Moreover, from 2020 onwards, the human coronaviruses (OC43, 229E, HKU1 and NL63) and SARS-CoV-2 virus were added to the diagnostic panel. These results were not included in this study to equalise the odds of co-infection between periods. The paediatric subjects were stratified into five groups, including infants (<1 y), toddlers (1 y to <3 y), preschool (3 y to <6 y), grade-schoolers (6 y to <12 y), and adolescents (12 y to <18 y).

## 3. Results

**Figure 1:**
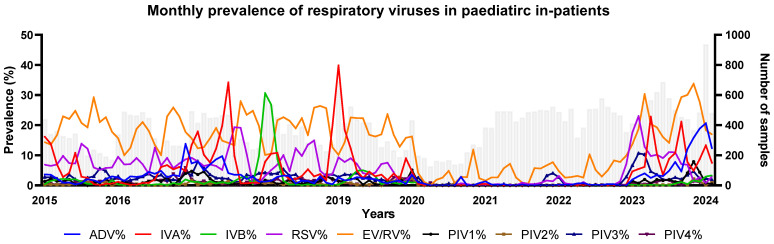
Prevalence of respiratory virus in paediatric in-patients before and after the SARS-CoV-2 pandemic. Monthly prevalence of detected respiratory viruses (left y-axis) with respective number of tested samples (columns in grey depicted by the right y-axis) from January 2015 to February 2024. AdV = adenovirus; IVs = influenza viruses; EV/RV = enterovirus/rhinovirus; RSV = respiratory syncytial viruses; PIVs = parainfluenza viruses.

We analysed 18,737 and 6001 respiratory specimens with a complete panel of virus PCR tests from the pre-COVID-19 and post-COVID-19 mask mandate periods, respectively (Table 1). Before the pandemic, EV/RV, influenza, and RSV represented the major burdens on paediatric admissions. During the COVID-19 lockdown, most respiratory virus infections were abolished while sporadic spikes of EV/RV and a minimal occurrence of AdV remained (Figure 1). A gradual rebound of viral infections was observed in the post-COVID-19 mask mandate period, with notable increases in AdV and EV/RV prevalence (Table 1).

The mean age of hospitalised patients increased from 3.49 y ± 0.03 y before COVID-19 to 4.37 y ± 0.05 y after the COVID-19 mask mandate (*p* < 0.001, Table 2). Generally, the rate of single-virus infection was significantly higher in the post-COVID-19 mask mandate period (48.36% vs. 43.00%, *p* < 0.0001) (Table 2). An age-group stratified analysis revealed that AdV had a remarkably escalated odds ratio for all age groups (OR: 4.53, 4.03, 2.32, 2.46, 1.31); meanwhile, the odds ratio decreased as the age increased. In addition, for RSV, there was a staggering increase in the odds ratio in older children, especially in 6 y to <12 y (OR: 3.38, *p* < 0.001), despite RSV typically contributing to the hospitalisation of children below five years old.

More importantly, the odds ratio of having more than one virus detected was higher in the post-COVID-19 era, particularly in the 1 y to <3 y (OR: 2.97, *p* < 0.001), 3 y to <6 y (OR: 3.05, *p* < 0.001), and 6 y to <12 y (OR: 2.70, *p* < 0.001) age groups. The number of co-infection cases (n = 298) and the detection rate (4.96%) in the one-year post-COVID-19 mask mandate exceeded the average of the four-year pre-COVID-19 era (n = 97, 2.07%, *p* < 0.001). Before COVID-19, EV/RV accounted for 68.4% of all viral co-infections, followed by RSV (35.4%), IAV (25.3%) and AdV (22.7%). Meanwhile, in the post-COVID-19 mask mandate period, EV/RV was still the predominant cause of viral co-infections (75.2%), with a notable increase in AdV (52.3%), followed by RSV (27.9%) and IAV (18.8%).

## 4. Discussion

Our study reported a rebound of respiratory viruses and increased multiple-virus infections after lifting the COVID-19 restrictions. We showed the changing patterns in AdV and RSV infections, affecting young and older children, respectively. Most of the increased vulnerability to respiratory viruses in children was seen in patients aged 3 y to <6 y. This could be attributed to the lack of exposure to viruses during lockdown, which is essential for building up immunity in early childhood [2,3].

The increase in virus detection post-COVID-19 mask mandate may also contribute to the immune suppression caused by prior SARS-CoV-2 infection in children. However, the causation is undiscernible as COVID-19 infection in children is mostly asymptomatic and does not require hospitalisation [4]. Therefore, it is important to monitor the trends and patterns of respiratory virus circulation and co-infection among children, especially those with underlying conditions or an immunocompromised status, and to provide timely and appropriate preventive and therapeutic interventions, as the increase in the co-detection of viruses may indicate worse clinical outcomes [5].

Further studies are needed to elucidate the causal relationship between SARS-CoV-2 exposure and subsequent respiratory virus susceptibility, as well as the long-term effects of interrupted virus exposure on children’s immune development. One limitation of this study is the short observation period. The yearly prevalence of IBV varied depending on the seasonality of strains and their circulation. The drastic reduction in IBV prevalence is likely due to IAV predominance in 2023–2024. Other confounding factors include differences in schools regarding the timing of reopening across grades and the lack of vaccination history within the cohort. Multiple attempts were made to study the effect of respiratory virus infection after the COVID-19 pandemic [6]. Despite these limitations, this single-centred observational study provides insights into the impact of a zero-COVID policy in paediatric settings via an age-stratified analysis, highlighting the major reservoir within the community. While the acute effect may diminish with the lifting of lockdown measures, the long-term effects remain elusive as certain viruses such as EV/RV and RSV are associated with the chronic development of asthma in children.

In conclusion, our findings suggest that the public health measures used to contain COVID-19 may have unintended consequences on children’s natural exposure and immunity to other respiratory viruses, potentially increasing morbidity and mortality in the post-pandemic era. This study provides crucial insights into the shifting landscape of paediatric respiratory infections in the post-COVID-19 era. By analysing a large dataset of respiratory specimens from pre- and post-COVID-19 mask mandate periods, this study highlights significant changes in the prevalence and patterns of viral infections among children. The findings underscore the unintended consequences of public health measures, such as mask mandates, on children’s natural exposure and immunity to respiratory viruses. Notably, this study reveals an increased incidence of multiple-virus infections and a shift in the age distribution of hospitalised patients, with older children showing higher susceptibility to certain viruses like RSV. These results emphasise the need for ongoing surveillance and tailored public health strategies that mitigate the long-term impacts of the pandemic on paediatric health. The study’s age- and sex-matched design provides a valuable framework for future research and clinical practice regarding the management of paediatric respiratory infections.

## Figures and Tables

**Table 1:** Demographics of study participants in the pre- and post-COVID-19 periods and the overall odd ratios of detectable agents in the post-COVID-19 period in children.

Demographics	Pre-COVID-19		COVID-19 Lockdown		Post-COVID-19 Mask Mandate		*p* Value#
**Duration**	**Jan 2015 to Dec 2019**		**Jan 2020 toJan 2024**		**Mar 2023 to Feb 2024**		
**Total specimen**	18,737		6358		6001		
**Age (Mean ± SEM)**	3.49 ± 0.03		4.40 ± 0.03		4.37 ± 0.05		<0.0001
<1	3942 (21.04%)		1518 (23.88%)		1012 (16.86%)		-
1 to <3	6010 (32.08%)		1931 (30.37%)		1341 (22.35%)		-
3 to <6	4535 (24.20%)		1313 (20.65%)		1849 (30.81%)		-
6 to <12	3301 (17.62%)		929 (14.61%)		1315 (21.91%)		-
12 to <18	949 (5.07%)		667 (10.49%)		484 (8.07%)		-
	n	%	n	%	n	%	
**Male**	10,538	56.00	3513	55.25	3416	56.92	0.362
**AdV%**	588	3.14	67	1.05	521	8.68	<0.0001
**IAV%**	1685	8.99	67	1.05	643	10.71	<0.0001
**IBV%**	598	3.19	11	0.17	52	0.86	<0.0001
**RSV%**	1406	7.50	276	4.34	462	7.70	0.502
**EV/RV%**	3403	18.16	815	12.82	1399	23.31	<0.0001
**PIV1%**	280	1.49	36	0.57	134	2.23	<0.0001
**PIV2%**	108	0.58	12	0.19	33	0.49	1
**PIV3%**	555	2.96	158	2.49	213	3.50	0.009
**PIV 4%**	235	1.25	21	0.33	53	0.88	0.004
**Whatever positive**	8450	45.10	1371	21.56	3200	53.32	<0.0001
**Single infection**	8063	43.00	1282	20.16	2902	48.36	<0.0001
**Co-infection**	387	2.07	89	1.40	298	4.96	<0.0001

**Table 2:** The odd ratios of detectable agents in the post-COVID-19 period with age stratification.

Age Range (Years Old)	<1	1 to <3	3 to <6	6 to <12	12 to <18
**A. Virus Detected**					
Pre-COVID-19	44.40%	49.50%	48.70%	38.10%	26.80%
Post-COVID-19	39.90%	58.60%	63.60%	51.10%	34%
Odds Ratio	0.83	1.44	1.84	1.70	1.38
95% CI	0.72–0.96	1.28–1.63	1.65–2.06	1.49–1.93	1.09–1.75
*p* Value	**0.010**	**<0.001**	**<0.001**	**<0.001**	**0.010**
**B. Single Virus Detected**					
Pre-COVID-19	42.20%	46.90%	46.50%	37.10%	26.40%
Post-COVID-19	36.50%	51.20%	57.10%	48.30%	32.20%
Odds Ratio	0.79	1.19	1.53	1.59	1.32
95% CI	0.68–0.91	1.06–1.34	1.37–1.71	1.39–1.80	1.04–1.68
*p* Value	**0.0010**	**0.0050**	**<0.001**	**<0.001**	**0.026**
**C. Multiple Viruses Detected**					
Pre-COVID-19	2.30%	2.60%	2.20%	1.10%	0.30%
Post-COVID-19	3.50%	7.50%	6.50%	2.80%	1.20%
Odds Ratio	1.55	2.97	3.05	2.70	3.96
95% CI	1.04–2.31	2.29–3.83	2.33–3.99	1.69–4.31	0.98–15.90
*p* Value	**0.032**	**<0.001**	**<0.001**	**<0.001**	0.069
**D. Adenovirus**					
Pre-COVID-19	0.80%	2.70%	5.50%	4.10%	1.30%
Post-COVID-19	3.40%	9.90%	12.00%	9.50%	1.70%
Odds Ratio	4.53	4.03	2.32	2.46	1.31
95% CI	2.76–7.44	3.17–5.11	1.92–2.80	1.91–3.17	0.53–3.23
*p* Value	**<0.001**	**<0.001**	**<0.001**	**<0.001**	0.64
**E. Influenza A virus**					
Pre-COVID-19	5.90%	8.80%	10.50%	11.10%	8.30%
Post-COVID-19	4.90%	9.10%	10.50%	14.80%	16.70%
Odds Ratio	0.84	1.03	1.00	1.39	2.21
95% CI	0.61–1.14	0.84–1.27	0.84–1.20	1.56–1.68	1.59–3.08
*p* Value	0.29	0.75	0.96	**0.001**	**<0.001**
**F. Influenza B virus**					
Pre-COVID-19	1.10%	2.20%	4.00%	6.10%	4.20%
Post-COVID-19	0.90%	0.60%	0.80%	0.80%	1.90%
Odds Ratio	0.78	0.27	0.20	0.13	0.43
95% CI	0.38–1.60	0.13–0.55	0.12–0.33	0.07–0.24	0.21–0.90
*p* Value	0.61	**<0.001**	**<0.001**	**<0.001**	**0.021**
**G. Respiratory Syncytial Virus**					
Pre-COVID-19	13.20%	10.40%	4.80%	1.10%	0.70%
Post-COVID-19	12.30%	8.70%	8.90%	3.50%	2.10%
Odds Ratio	0.92	0.82	1.95	3.38	2.83
95% CI	0.74–1.13	0.67–1.01	1.58–2.41	2.17–5.28	1.07–7.51
*p* Value	0.433	0.071	**<0.001**	**<0.001**	**0.038**
**H. Enterovirus/ Rhinovirus**					
Pre-COVID-19	17.50%	20.50%	20.10%	14.10%	11.20%
Post-COVID-19	14.40%	28.80%	29.70%	20.20%	11.00%
Odds Ratio	0.80	1.57	1.68	1.54	0.98
95% CI	0.66–0.96	1.37–1.80	1.49–1.90	1.30–1.81	0.69–1.39
*p* Value	**0.021**	**<0.001**	**<0.001**	**<0.001**	0.93
**I. Parainfluenza virus 1**					
Pre-COVID-19	1.60%	1.70%	1.80%	0.90%	0.10%
Post-COVID-19	1.20%	2.70%	2.90%	2.40%	0%
Odds Ratio	0.73	1.58	1.63	2.72	-
95% CI	0.39–1.35	1.08–2.32	1.15–2.31	1.65–4.49	-
*p* Value	0.39	**0.026**	**0.0070**	**<0.001**	1
**J. Parainfluenza virus 2**					
Pre-COVID-19	0.50%	0.40%	0.80%	0.80%	0.40%
Post-COVID-19	0.10%	0.30%	0.40%	1.40%	0.40%
Odds Ratio	0.19	0.78	0.54	1.82	0.98
95% CI	0.023- 1.4	0.27–2.3	0.25–1.2	0.99–3.4	0.18–5.4
*p* Value	0.104	0.806	0.134	0.061	1
**K. Parainfluenza virus 3**					
Pre-COVID-19	4.80%	4.40%	1.70%	0.50%	0.80%
Post-COVID-19	5.50%	5.40%	3.60%	1.20%	0.60%
Odds Ratio	1.15	1.25	2.17	2.25	0.73
95% CI	0.85–1.56	0.95–1.63	1.56–3.03	1.42–4.42	0.19–2.78
*p* Value	0.37	0.11	**<0.001**	**0.021**	0.76
**L. Parainfluenza virus 4**					
Pre-COVID-19	1.50%	1.20%	1.90%	0.50%	0%
Post-COVID-19	0.80%	1.00%	1.40%	0.30%	0.40%
Odds Ratio	0.53	0.85	0.72	0.56	-
95% CI	0.25–1.12	0.48–1.50	0.46–1.13	0.19–1.65	-
*p* Value	0.12	0.68	0.17	0.35	0.11

Fisher’s exact test and t-test were used to investigate the differences in sex and age between the pre- and post-COVID-19 mask mandate period, respectively. Fisher’s exact test was used to study the detectable agents in the post-COVID-19 period stratified by age groups. A two-sided *p* value < 0.05 was considered significant. The colour gradient reflects the increase (red) or decrease (blue) in OR for detecting the virus in that specific age group post-COVID-19 mask mandate period.

## Data Availability

Data is unavailable due to ethical restrictions.

## References

[1] Shen X.-R., Geng R., Li Q., Chen Y., Li S.-F., Wang Q., Min J., Yang Y., Li B., Jiang R.-D. (2022). ACE2-independent infection of T lymphocytes by SARS-CoV-2. Signal Transduct. Target. Ther..

[2] Billard M.-N., Bont L.J. (2023). Quantifying the RSV immunity debt following COVID-19: A public health matter. Lancet Infect. Dis..

[3] Messacar K., Baker R., Park S., Nguyen-Tran H., Cataldi J., Grenfell B. (2022). Preparing for uncertainty: Endemic paediatric viral illnesses after COVID-19 pandemic disruption. Lancet.

[4] Chua G.T., Wong J.S.C., Lam I., Ho P.P.K., Chan W.H., Yau F.Y.S., Duque J.S.R., Ho A.C.C., Siu K.K., Cheung T.W. (2021). Clinical characteristics and transmission of COVID-19 in children and youths during 3 waves of outbreaks in Hong Kong. JAMA Netw. Open.

[5] Feldman C., Anderson R. (2021). The role of co-infections and secondary infections in patients with COVID-19. Pneumonia.

[6] Maltezou H.C., Papanikolopoulou A., Vassiliu S., Theodoridou K., Nikolopoulou G., Sipsas N.V. (2023). COVID-19 and Respiratory Virus Co-Infections: A Systematic Review of the Literature. Viruses.

